# Dysregulated cholesterol regulatory genes in hepatocellular carcinoma

**DOI:** 10.1186/s40001-023-01547-z

**Published:** 2023-12-09

**Authors:** Dan Cao, Huan Liu

**Affiliations:** 1 Department of Respiratory and Critical Care Medicine, Affiliated Hospital of North Sichuan Medical College, No. 1 the South of Maoyuan Road, Nanchong, 637000 Sichuan People’s Republic of China; 2 Center of Infectious Diseases, West China Hospital of Sichuan University, Chengdu, 610041 China

**Keywords:** Hepatocellular carcinoma, Cholesterol, Tumorigenesis, Therapy

## Abstract

Cholesterol is an indispensable component in mammalian cells, and cholesterol metabolism performs important roles in various biological activities. In addition to the Warburg effect, dysregulated cholesterol metabolism is one of the metabolic hallmarks of several cancers. It has reported that reprogrammed cholesterol metabolism facilitates carcinogenesis, metastasis, and drug-resistant in various tumors, including hepatocellular carcinoma (HCC). Some literatures have reported that increased cholesterol level leads to lipotoxicity, inflammation, and fibrosis, ultimately promoting the development and progression of HCC. Contrarily, other clinical investigations have demonstrated a link between higher cholesterol level and lower risk of HCC. These incongruent findings suggest that the connection between cholesterol and HCC is much complicated. In this report, we summarize the roles of key cholesterol regulatory genes including cholesterol biosynthesis, uptake, efflux, trafficking and esterification in HCC. In addition, we discuss promising related therapeutic targets for HCC.

## Introduction

Primary liver cancer is one of the most common malignant tumors and the leading cause of cancer-related deaths worldwide. According to GLOBOCAN 2020, it is predicted that there are near 930,000 new cases of primary liver cancer and 800,000 deaths, respectively [1]. Hepatocellular carcinoma (HCC) is the most prevalent type of primary liver tumor, which poses a serious threat to human health.

Cholesterol is an indispensable component of plasma membranes and plays a key role in maintaining permeability and fluidity of cytomembrane [2]. In addition to its structural support for cell membranes, it is an essential component of lipid rafts and plays an important role in intercellular signaling [3]. Hepatoma cells require vigorous cholesterol metabolism to synthesize plasma membranes and perform other functions due to their rapid growth. Therefore, exploring the relationship between genes involved in cholesterol metabolism and HCC is of great significance for understanding the molecular mechanism of HCC.

## Overview of cholesterol metabolism

**Fig. 1:**
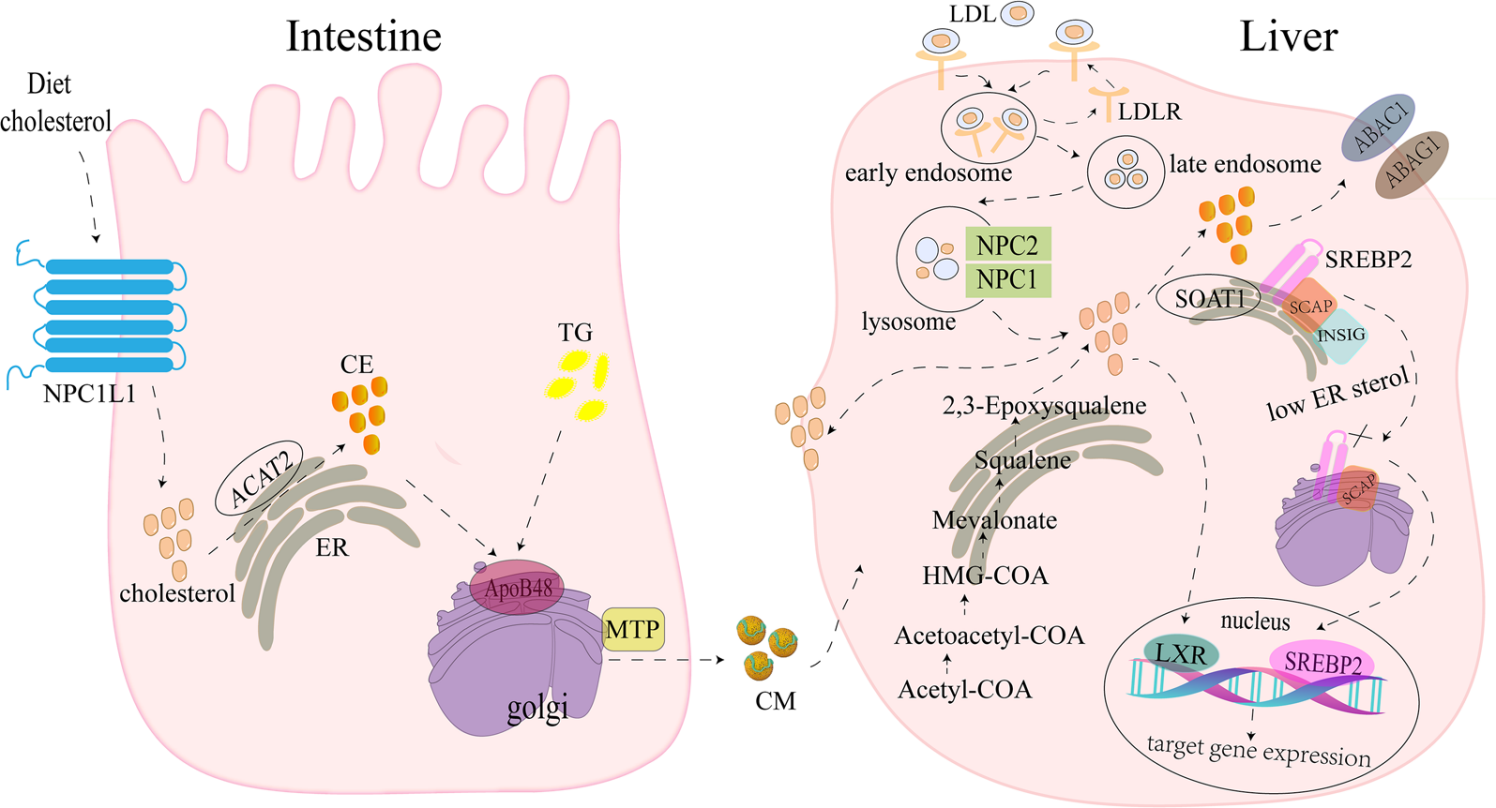
Schematic illustration of cholesterol metabolism homeostasis

The homeostasis of cholesterol metabolism is an important guarantee for the organism to exert its physiological function [4]. There are several processes for cholesterol metabolism, including cholesterol biosynthesis, uptake, efflux, trafficking and esterification. Cholesterol is mostly dependent on cell synthesis. The endogenous biosynthesis pathway converts acetyl-CoA (CoA) to cholesterol through nearly 30 enzymatic reactions, including mevalonate pathway, squalene biosynthesis and transformation. Within these enzymatic reactions, HMG-CoA reductase (HMGCR) and squalene epoxidase (SQLE) are two key rate-limiting enzymes that control the rate-limiting steps of the conversion of HMG-CoA to MVA and the catalytic conversion of squalene to 2-epoxy squalene, respectively [5]. Blood cholesterol enters cells mainly via LDL receptor (LDLR)-mediated endocytosis. LDL is isolated from LDLR in endosome and further transferred to lysosome [6]. Free cholesterol was released from lysosome and formed cholesterol ester mediated by sterol O-acyltransferase (SOAT) [7]. Moreover, Niemann–Pick C1-like 1 (NPC1L1) facilitates uptake of dietary cholesterol by small intestinal cells [8]. Transcription factor sterol regulatory element binding protein (SREBP) plays a key role in regulating cholesterol homeostasis [9]. Among them, SREBP2 plays a major regulatory role. When the concentration of cholesterol on the endoplasmic reticulum membrane increases, the SREBP cleavage activating protein (SCAP), as a cholesterol sensitive protein, changes its conformation and binds to the endoplasmic reticulum anchoring protein INSIG, thereby retaining the SCAP/SREBPs complex on the endoplasmic reticulum. When the cholesterol level of endoplasmic membrane decreases, insulin-induced gene (INSIG) was isolated from SCAP and degraded by proteasome, releasing SCAP/SREBPs complex to Golgi complex, in which SREBPs was activated by S1P and S2P. The N-terminal domain of SREBPs enters the nucleus and activates gene transcription required for cholesterol synthesis and uptake. Excess cholesterol is not only stored in lipid droplets as cholesterol esters, but also transported into the bloodstream via the ATP-binding cassette transporter A1 (ABCA1) and ATP-binding cassette transporter G1(ABCG1) [2, 10]. In short, intracellular cholesterol levels are precisely controlled by biosynthesis, uptake, efflux, trafficking and esterification. Problems with any of processes lead to an imbalance of intracellular cholesterol (Fig. 1).

## Gene involved in cholesterol metabolism in hepatocellular carcinoma

**Fig. 2:**
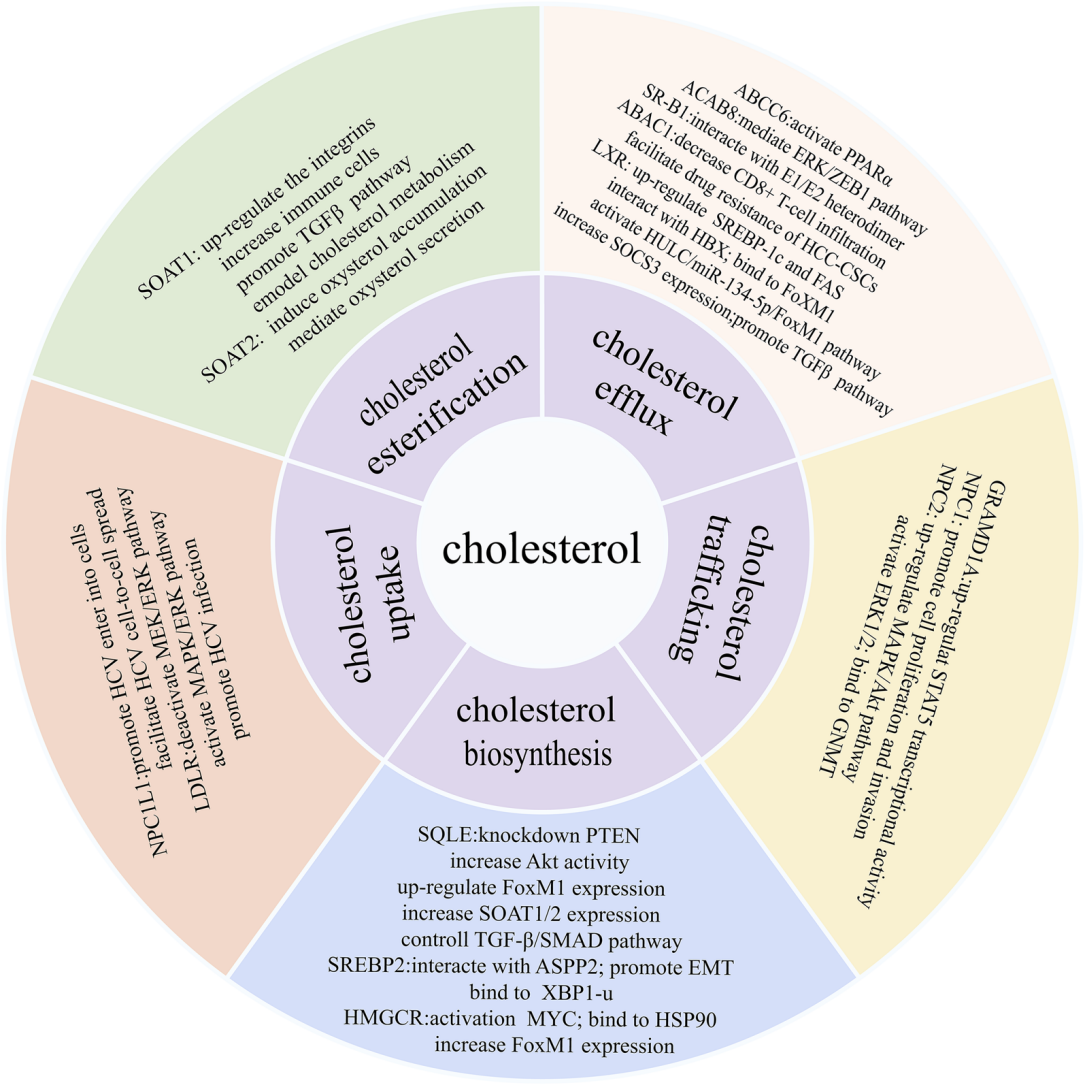
Molecular mechanism of cholesterol regulatory genes in HCC

Several aberrantly expressed genes regulate cholesterol metabolic changes that prompt cell proliferation, migration, and invasion in HCC (Fig. 2). As a result, developing strategies to target these genes might offer the foundation for innovative treatment alternatives.

### Cholesterol biosynthesis

#### HMG-CoA reductase (HMGCR)

The carcinogenic effect of HMGCR has been reported in many tumors, such as gastric cancer, ovarian cancer and breast cancer [11]. HMGCR was higher in HCC and positively correlated with poor prognosis in patients with HCC [12, 13]. MYC is an underlying target for several malignancies. Interfering with HMGCR inhibited HCC growth by activation of MYC, which suggested that HMGCR might be an effective target for the treatment of HCC [14]. Heat shock protein 90 (HSP90) promoted proliferation and inhibited apoptosis of HCC cell, but its mechanism was not clear. Li et al. confirmed that HSP90 promoted the proliferation and migration of HCC cell by increasing the expression of HMGCR [15]. Forkhead Box M1 (FoxM1) was recognized as a factor that promoted the development and progression of HCC. One study showed that HMGCR-inhibition increased HCC cell death via decreasing the expression and the transcriptional activity of FoxM1 [16].

#### Squalene epoxidase (SQLE)

As the second rate-limiting enzyme of cholesterol synthesis, cholesterol metabolism disorders caused by the dysregulation of SQLE expression are closely related to tumor development, which are involved in various processes such as tumor proliferation, apoptosis, epithelial mesenchymal transition and metastasis [17, 18]. The upregulation of SQLE expression was associated with advanced HCC histological grade, advanced AJCC stage, elevated α-fetoprotein and poor clinical outcome [19–21]. Liu et al. found that SQLE increased the expression of SOAT1/2 to promote intracellular cholesteryl ester synthesis, which in turn accelerated HCC cells growth [19]. SQLE also increased Akt activity by knockdown PTEN. Specifically, the conversion of squalene to 2,3-epoxy squalene catalyzed by SQLE required consumption of NADPH, and the NADPH deficiency induced oxidative stress in HCC cells, which upregulation of DNMT3A expression leaded to PTEN silence and contributed to Akt–mTOR pathway activation [19]. P53, the most common mutant gene in malignancies, regulates multiple functions such as apoptosis, cell cycle arrest and senescence. P53 repressed HCC growth by directly inhibiting SQLE transcription and reducing cholesterol synthesis. SQLE over-expression accelerated HCC growth, which further indicated that SQLE played an important role in p53-mediated tumor suppression [22]. In addition, SQLE promoted the development of HCC by activating TGFβ/SMAD pathway [23]. The above studies suggested that SQLE might be a potential therapeutic target for HCC.

#### Sterol regulatory element-binding protein 2 (SREBP2)

SREBP2 is engaged in the regulation of genes involved in cholesterol homeostasis. Moon et al. found that p53 also regulates SREBP2. Specifically, P53 inhibited the mevalonate pathway by preventing SREBP2 maturation, ultimately slowing down the progression of HCC [24]. ASPP2 interacted with p53 and stimulated the p53-mediated anti-tumor effects. Bai et al. found that the anti-tumor activity of ASPP2 was closely related to SREBP2. They proved that ASPP2 interacted with SREBP2 in the nucleus and reduced the transcriptional activity of SREBP2, which resulted in the down-regulation of the expression of the key enzymes in the mevalonate pathway, ultimately inhibiting the progression of HCC [13]. Chen et al. indicated that FASN deletion promoted nuclear localization and activation of SREBP2, which triggered de novo lipogenesis and cholesterol biosynthesis, eventually leading to hepatocarcinogenesis [25]. Epithelial–mesenchymal transition (EMT) plays an important role in the heterogeneity and tumor metastasis. SREBP2 promoted HCC cells invasion and metastases by inducing EMT [26]. Moreover, recent studies have shown that XBP1-u promoted tumorigenesis. Mechanically, XBP1-u colocalized with SREBP2 and suppressed its ubiquitin/proteasome degradation, leading to cholesterol synthesis and lipid accumulation, finally inducing liver carcinogenesis [27].

### Cholesterol uptake

#### Niemann–Pick C1-like 1 (NPC1L1)

NPC1L1, a membrane protein with 1332 amino acids, is abundantly expressed in the small intestine and liver. It plays an important role in intestinal absorption of cholesterol. Most of the studies about NPC1L1 in tumors focus on clinical research and bioinformatics analysis. Chen et al. found that HCC patients with a relatively low expression level of NPC1L1 had a poor clinical outcome and were more prone to occur HCC recurrence [28]. HCV infection is a major risk for the development of HCC. NPCL1 not only contributed to HCV enter into cells, but also promoted HCV cell-to-cell spread, might contributing to development of HCV-HCC [29]. Drug-resistant recurrence is a major challenge in treatment of tumor. Zhang et al. found that NPC1L1 was closely associated with drug-tolerant persister state of tumor cells. Inhibition of NPC1L1 induced oxidative stress‐mediated cell death and disrupted adaptive responses of drug-tolerant persister cells to chemotherapy [30]. The molecular mechanism of NPC1L1 in HCC has not yet been reported. Whether the abnormal expression of NPC1L1 will induce drug resistance in HCC cells, it seems to be worth exploring.

#### Low-density lipoprotein receptor (LDLR)

LDLR is a transmembrane glycoprotein located on the cell membrane. Its binding to LDL promotes cholesterol uptake by endocytosis. The expression level and prognostic value of LDLR varies in different types of tumors. The high expression of LDLR indicated poor prognosis in patients with ovarian cancer [31]. In renal cancer tissues, LDLR expression was higher than in normal renal tissues. Knockdown LDLR inhibited renal cancer cell proliferation and induced cell cycle arrest [32]. LDLR was considered as an essential co-receptor for the HCV entry into cells. Knockdown LDLR hindered HCV infection [33]. Moreover, another study demonstrated that LDLR regulated the activation of MAPK/ERK pathway triggered by HCV E2, thereby maintaining the growth and survival of Huh-7 cells [34]. These resulted showed that LDLR might played an important role in progression of HCV-HCC. Mamatha et al. found that the expression of LDLR expression was higher in HCC tissues, which contributed to the uptake of exogenous cholesterol in HCC [35]. In contrast, Chen et al. observed that the expression level of LDLR was lower in HCC samples. The lower LDLR expression might be an indicator of poor clinical outcome in HCC patients. Silence LDLR activated MEK/ERK pathway and facilitated cholesterol synthesis, ultimately promoting HCC cell proliferation and metastasis [36]. The difference of findings might be attributed to the strong heterogeneity of HCC in different studies. The molecular mechanisms of LDLR in HCC still need to be explored.

### Cholesterol trafficking

#### Niemann–Pick type C (NPC)

The researches of NPC1 in HCC focus on bioinformatics analysis. Based on analysis of ICGC and TCGA databases, the higher NPC1 suggested unfavorable prognosis of HCC patients. The results of proteomic analysis also showed that NPC1 expression was upregulated in HCC tissues [37, 38]. Du et al. found that NPC1 inhibited the proliferation and metastasis of Huh7 cells in vitro, but the mechanism has not been reported [39]. NPC2, a small secretory glycoprotein, is widely located on lysosomal compartment. The HCC patients with lower NPC2 had higher α-fetoprotein, later histological stage and poorer prognosis. NPC2 silence promoted HCC cell proliferation, migration and xenograft tumorigenesis by regulating ERK1/2 activation [40]. Meanwhile, a study reported that NPC2 knockdown attenuated the therapeutic effect of sorafenib by activating MAPK/AKT signal pathway in HCC cells [41]. Moreover, other study reported that NPC2 interaction with GNMT triggered cholesterol accumulation, which might may provide novel therapeutic strategies for HCC [42].

#### GRAM structural domain-containing protein 1A (GRAMD1A)

GRAMD family proteins consist of GRAMD1A, GRAMD1B, GRAMD1C, GRAMD2, and GRAMD3, of which GRAMD1A, GRAMD1B, and GRAMD1C mediate cholesterol transport from plasma membrane to endoplasmic reticulum. Fu et al. found that the expression of GRAMD1A was increased in HCC tissues, and its higher expression was positively associated with unfavorable clinical outcome of HCC patients [43]. Mechanically, GRAMD1A accelerated self-renewal of HCC stem cells and progression of HCC through upregulating STAT5 transcriptional activity [43].

### Cholesterol efflux

#### Liver X receptors (LXRs)

LXRs, including LXRα and LXRβ isoforms, performs a critical function in maintenance intracellular cholesterol homeostasis. LXR is activated by LXR agonists, and then LXR combines with retinoid X receptor (RXR) to form a heterodimer. The LXR–RER heterodimer binds to LXR-responsive element (LXRE) and regulates the expression of genes involved in cholesterol metabolism [44]. Hepatitis virus infection and liver steatosis are the most main risk factors for HCC. Na et al. found that LXR interacted with HBV X protein (HBx) in the nucleus and enhanced the transactivation function of LXR, inducing upregulation of SREBP-1c and FAS to accelerate lipid droplets accumulation, finally resulting in HBV-associated hepatic carcinogenesis [45]. Kin et al. also observed similar results [46]. HCV core protein might contribute to hepatic steatosis and HCV replication through LXR-regulated lipogenesis [47]. One study demonstrated that HCV core protein promoted the binding LXR between LXRE to activate SREBP-1c promoter activity, finally facilitating occurrence of hepatic steatosis and HCC [48]. Bakiri et al. proved that c-Fos down-regulated expression and activity of LXRα, resulting in alteration of hepatocyte morphology, infiltration of immune cells and formation of necrotic foci, ultimately promoting proliferation, dedifferentiation and DNA damage [49]. FoxM1 is related to progression of HCC by increasing the expression of cell cycle genes such as cyclin D1 and cyclin B1. Hu et al. found that LXRα binds to an inverted repeat IR2 in the promoter region of FoxM1 gene and decreased the expression of FoxM1, cyclin D1 and cyclin B1, thereby inhibiting proliferation and growth of HCC cells [50]. He et al. demonstrated that LXRα upregulated miR-134-5p while down-regulate FoxM1 by decreasing HULC, eventually suppressing HCC cells growth [51]. Moreover, LXRα increased expression of SOCS3 by enhancing the mRNA stability, then suppressing cyclin D1 and elevating p21 and p27, finally leading to cell cycle arrest and inhibiting HCC cells growth [52]. The upregulation of LXRα reduced TGFβ-induced Snail expression, leading to suppressing mesenchymal differentiation, the generation of reactive oxygen and promoting apoptotic response [53]. Morén et al. demonstrated that LXRα activation suppressed TGFβ-induced differentiation of cancer-associated fibroblasts by repressing the promoter activity of ACAT2, resulting in limiting HCC growth [54]. Lin et al. observed that LXRα upregulated the transcription level of miRNA-378a-3p and enhanced anti-tumor efficacy of sorafenib [55]. Drug induced lipotoxicity might be a potential therapeutic strategy for the treatment of HCC. A research demonstrated that LXRα activation and Raf inhibition leaded to accumulation of toxic saturated fatty acid, inducing the apoptosis of HCC cells [56]. Furthermore, LXR might serve as a marker for HCC prognosis. Long et al. found that LXR was much lower in HCC. The 5-year survival rate of patients with low LXR expression was lower than that of patients with high LXR expression [57].

#### ATP-binding cassette (ABC) transporters

ATP-binding cassette (ABC) transporters include a large family of membrane-bound proteins, some of which are associated with cholesterol efflux. ABC transporters are divided into seven subfamilies, known as ABCA-ABCG. Li et al. observed that ABCA1, cholesterol efflux transporter, upregulated in tumor monocytes/macrophages, thereby resulting in the production of immature and immunosuppressive monocytes/macrophages. High numbers of ABCA1 + monocytes/macrophages in HCC decreased CD8 + T cell infiltration, consequently leading to an unfavorable prognosis for HCC [58]. Cancer stem cells (CSCs) play a vital role in mediating unrestrained cell proliferation and chemoresistance. Hu et al. demonstrated that the over-expression of ABCA1 facilitated drug resistance of Lgr5 + HCC-CSCs cells to doxorubicin [59]. Xi et al. proved the higher ABCG1 predicted poor prognosis in patients with HCC [60]. One study demonstrated that ABCG1 knockdown leaded to decreased oxaliplatin resistance, indicating that that it played an important role in acquiring drug resistance of HCC cells [61]. Liao et al. observed similar result that ABCG1 silence reversed the oxaliplatin resistance in HCC [62]. Zhao et.al found that the down-regulation of ABCC6 accelerated cell proliferation, suppressed cell cycle arrest and cell apoptosis by deactivating PPARα [63]. Additionally, Researchers also found that the low expression of ACAB8 contributed to epithelial–mesenchymal transition by mediating ERK/ZEB1 pathway, consequently promoting proliferation and metastasis of HCC [64].

#### Scavenger receptor B1 (SR-B1)

SR-B1, widely expressed in liver and steroidogenic cells, promoted cholesterol efflux from peripheral tissues, such as macrophages back to liver. Higher SR-B1 expression has been recognized as a biomarker of cancer progression, such as melanoma and lung cancer [65, 66]. The SR-B1 interaction with E1/E2 heterodimer triggered HCV enter into liver cell by endocytosis [67]. However, the molecular mechanism of SR-B1 in HCC needs to be further explored.

### Cholesterol esterification

#### Sterol O-acyltransferase (SOAT)

SOAT, also known as acyl coenzyme A cholesterol acyltransferase (ACAT), is a membrane-bound enzyme that promotes esterification of cholesterol and fatty acids into cholesterol esters. Two isoforms of SOAT have been identified, SOAT1 and SOAT2. SOAT2 is predominantly found in fetal hepatocytes and intestinal epithelial cells, while SOAT1 is widely expressed in most cells [6]. The over-expression of SOAT1 has been recognized as an indicator of tumor progression in glioblastoma, pancreatic cancer, and prostate cancer [68–70].The high expression of SOAT1 was positively correlated with poor prognosis of HCC patients [71]. Jiang et al. found that SOAT1 knockdown inhibited growth and migration of HCC via down-regulating the integrins and TGFβ signal pathway [72]. Reprogramming energy metabolism is a hallmark of cancer [73]. Wang et al. demonstrated that target SOAT1 remodeled cholesterol metabolism and enhanced immune cells to suppress HCC tumor growth [74]. The P53 plays an important role in mediating lipid metabolism and energy generation. Zhu et al. proved that P53 deficiency upregulated the expression of SOAT1 and facilitated cholesterol esterification and fatty acid synthesis, consequently leading to HCC growth [75]. These results suggested that SOAT1 might be a potential target for P53-deficient HCC. In addition, Chen et al. analyzed the relationship between SOAT1 genetic variants and HCC. They found that SOAT1 rs10753191 and a haplotype TGA were related to decreased HCC risk [76]. SOAT2 is predominantly distributed in hepatocytes and intestinal epithelial cells. Liu et al. suggested SOAT2 induced oxysterol accumulation and mediated oxysterol secretion, leading to promoting HCC growth [77].

## Therapeutic insights into HCC

To date, many studies have suggested that abnormal cholesterol metabolism could regulate invasion and metastasis of HCC. Therefore, we have provided an overview of drugs targeting cholesterol regulatory genes in HCC (Table 1).

**Table 1:** Potential reagents for targeting cholesterol metabolism in HCC

Gene	Reagents	Mechanism and effects	References
HMG-CoA	Atorvastatin	Restore cholesterol-induced gut microbiome dysbiosis to inhibit lipid accumulation and HCC cell proliferation	[78]
		Inhibit YAP and Akt activation to decrease prognostic liver signature score	[79]
		Decrease MMP2 and MMP9 to prevent proliferation and invasiveness of HCC cells	[80]
		Suppress the IL-6/STAT3 pathway to induce cellular senescence in HCC cells	[81]
		Mediate TGFβ/pERK signal pathway to inhibit HCC cell proliferation and angiogenesis	[82]
		Target AMPK/p21 signaling pathway to induce autophagy of HCC cells	[83]
	Simvastatin	Alter the expression of cell cycle regulating proteins to induce apoptosis and cell cycle arrest	[84]
		Suppress STAT3/SKP2 pathway and activate AMPK to induce cell cycle arrest	[85]
		Induce HCC cells apoptosis and improve liver fibrosis and necroinflammatory score	[86]
		Upregulate Notch1 expression to induce growth inhibition and apoptosis	[87]
		Target HIF-1α/PPAR-γ/PKM2 axis to prevent cell proliferation and induce apoptosis, and re-sensitize HCC cells to sorafenib	[88]
SQLE	Terbinafine	Prevent Akt-mTOR signaling and upregulate PTEN expression to inhibit HCC growth	[19]
	NB-598	Down-regulate TGFβ expression and SMAD2/3 phosphorylation to inhibit the viability of HCC cells	[23]
SREBP2	Betulin	Thwart the glycolytic activity to inhibit tumor growth	[90]
		Target mTOR/IL-1β pathway to enhance the anti-tumor of lenvatinib	[91]
SOAT1	Avasimibe	Restrain the proliferation and migration of HCC cells	[72, 75]
		Disrupt lipid homeostasis to inhibit HCC growth	[92]
LXR	TO901317	Increase REPS2 expression to inhibit proliferation and migration of HCC cells	[98]
		Inhibit TGF-dependent CAF differentiation to restrict the progression of HCC	[54]
		Knockdown the expression of MET and EGFR to enhance the anti-cancer activity of sorafenib	[99]
	GW3965	Increase transcription level of miRNA-378a-3p to enhance anti-tumor efficacy of sorafenib	[55]
	Withaferin A	Activate LXRα and inhibit the transcriptional activity of NF-κB to suppress the proliferation, migration, invasion of HCC cells	[100]
		Affect Nrf2-mediated EMT and ferroptosis to prevent the metastasis and drug resistance in hepatoma cells	[102]

### Statins

Statins, inhibitor of HMG-CoA reductase, are commonly used in the treatment of hypercholesterolaemia for prevention of cardiovascular diseases. Several studies have shown that statins exert anti-tumor effects by inhibiting several processes such as cancer cell proliferation, invasion and tumor angiogenesis. Recently, Zhang et al. indicated that atorvastatin increased IPA and decreased TCA through reversing cholesterol-induced gut microbiome dysbiosis, thereby inhibiting lipid accumulation and HCC cell proliferation, ultimately preventing NAFLD–HCC development [78]. Kim et al. identified that atorvastatin inhibited YAP and Akt activation to decrease prognostic liver signature score [79]. Ghalali et al. also observed that atorvastatin deactivated Akt. They pointed out atorvastatin decrease pAkt, pGsk3β, and lipogenesis by targeting P2X-Akt signaling pathway [80]. Moreover, atorvastatin prevented proliferation and invasiveness of HCC cells by decreasing the expression of MMP2 and MMP9 [80]. Cellular senescence plays an important role in restriction tumor growth. One study demonstrated that atorvastatin decreased the expression of hTERT via deactivating the IL-6/STAT3 pathway, consequently inducing cellular senescence to prevent HCC growth [81]. Angiogenesis is involved in the development and pathogenesis of HCC. Atorvastatin showed antiproliferative and antiangiogenic effects in HCC via TGFβ/pERK signal pathway [82]. However, there are different results. Yang et al. proved that atorvastatin caused endoplasmic reticulum stress via target AMPK/p21 signaling pathway, consequently resulting in autophagy to promote survival of HCC cells. The result showed that combinations of atorvastatin with autophagic inhibitor provided a novel therapeutic strategy for HCC [83].

Simvastatin is another cholesterol-lowering medication and might be control cell cycle processes in HCC. Relja et al. observed that simvastatin altered the expression of cell cycle regulating proteins, leading to apoptosis and cell cycle arrest in HCC [84]. Wang et al. demonstrated that simvastatin induced cell cycle arrest by suppression of STAT3/SKP2 pathway and activation of AMPK in HCC [85]. Elleithi et al. proved that simvastatin induced HCC cells apoptosis and improved liver fibrosis and necroinflammatory score [86]. Huang et al. identified that simvastatin induced growth inhibition and apoptosis of HCC cells by upregulation of Notch1 expression [87]. Furthermore, simvastatin inhibited the HIF-1α/PPAR-γ/PKM2 axis, leading to prevent cell proliferation and induce apoptosis, and re-sensitizing HCC cells to sorafenib [88].

### SQLE inhibitor

Terbinafine, an inhibitor of SQLE, is a broad-spectrum antifungal drug. Recent evidence shows that terbinafine may have an anti-cancer efficacy. Liu et al. found that terbinafine inhibited HCC growth through prevention of Akt–mTOR signaling and restoration of PTEN expression [19]. NB-598, is a synthesized inhibitor of SQLE. In 1990, a published report suggested that NB-598 could inhibit cholesterol synthesis in HepG2 cells [89]. Another study demonstrated that NB-598 inhibited the viability of HCC cells by down-regulation TGFβ expression and SMAD2/3 phosphorylation [23].

### SREBP2 inhibitor

Betulin, an inhibitor of SREBP2, has been identified to inhibit the growth and survival of cancer cells. Yin et al. demonstrated that betulin significantly suppressed tumor growth via thwarting the glycolytic activity of HCC cells [90]. At the same time, the combination betulin and sorafenib enhanced the anti-tumor efficacy in HCC [90]. Additionally, another study suggested that betulin enhanced the anti-tumor effect of lenvatinib through targeting the mTOR/IL-1β pathway [91].

### Avasimibe

Avasimibe, a SOAT inhibitor and cholesterol-lowering medication, can inhibit the esterification of cholesterol. Accumulating studies showed that avasimibe had a good anti-cancer effect in various cancer types, such as cholangiocarcinoma, glioblastoma and prostate cancer. Recent research has shown that the SOAT1 inhibitor Avasimibe restrained the proliferation and migration of HCC cells [72, 75]. Moreover, the combination of avasimibe with etomoxir was more effective in suppressing HCC growth via disrupting lipid homeostasis [92]. In addition to its direct anti-tumor effects, researchers have also found the antiviral property of avasimibe. Hu et al. demonstrated that combination of avasimibe with direct-acting antivirals (DAAs) could impair the assembly of HCV virions and exist pan-genotypic inhibitory activity [93]. Furthermore, the avasimibe could enhance HBV-specific CTL immune responses by mediating cell membrane cholesterol contents [94]. Avasimibe can also act as an immunomodulatory medicine. Researcher has found that avasimibe could increase the plasma membrane cholesterol level of CD8 + T cells, promoting T cell receptor signaling and enhancing tumor-killing function of CD8 + T cells [95]. Zhao et al. proved that avasimibe enhanced the anti-cancer efficacy of CART cells [96]. In early clinical trials of atherosclerosis, avasimibe revealed a well-tolerated safety profile [97]. Therefore, a clinical trial of avasimibe may be considered in patients with HCC.

### LXR agonist

Several studies have revealed LXR agonist exhibited anti-cancer effects in various types of cancers. Recently, He et al. proved that TO901317 inhibited the proliferation and migration of HCC cells via increasing REPS2 expression at the transcriptional level [98]. Moreover, TO901317 inhibited TGF-dependent CAF differentiation, thereby restricting the progression of HCC [54]. Another study suggested that TO901317 strengthened the anti-cancer activity of sorafenib via knockdown the expression of MET and EGFR [99]. GW3965, another LXR agonist enhanced anti-tumor efficacy of sorafenib by increasing transcription level of miRNA-378a-3p [55]. Withaferin A, originated from Withania somnifera plant, could activate the activity of LXRα and thwart NF-κB transcriptional activity, consequently preventing the proliferation, migration and invasion of HCC cells [100]. Varsha et al. discussed how Withaferin A inhibits NF-κB by acting on LXRα. They proposed that LXRα got activated by Withaferin A and formed obligate heterodimers with RXR, and then control the expression of target genes containing LXRE. Several researches have reported that the target genes of NF-κB contained LXRE on their promoters. Therefore, Withaferin A inhibit target genes of NF-κB at transcription level by controlling the formation obligate heterodimers between LXRα and RXR [101]. Another study also identified that Withaferin A influenced the Keap1/Nrf2 signaling to mediate EMT and ferroptosis, eventually preventing the metastasis and drug resistance in hepatoma cells [102].

## Conclusions

Because of the rapid growth and high biosynthesis of cancers, cholesterol homeostasis imbalances are often observed in various types of cancers. In this review, we summarized the potential mechanisms of gene involved in cholesterol metabolism in HCC. The expression of most cholesterol metabolism related genes in HCC is increased (e.g., HMG-CoA, SQLE, SREBP2, SOAT1), its over-expression promotes proliferation, migration and invasion of hepatoma cells. However, the roles of some genes in HCC exert contentious effect such as LXR. This phenomenon may be associated with the extensive heterogeneity of HCC samples. Therefore, the molecular mechanism of cholesterol metabolism related genes in HCC needs further explore. Additionally, we reviewed the role of drugs that modulate cholesterol metabolism in HCC. Most of drugs that targeted cholesterol metabolism related genes to lower cholesterol levels showed anti-tumor activity in basic experiments. Some clinical studies also suggested that cholesterol-lowering drugs (e.g., statins) prolonged the survival of patients with advanced HCC. It is regrettable that there is lack of multicenter and large-sample clinical trials to prove the anti-tumor efficacy of cholesterol-lowering drugs. Its clinical application remains challenging. In the future, we may be able to design multicenter and large-sample clinical trials to evaluate the effects of these cholesterol-lowering drugs in HCC, aiming to provide more options for the treatment of HCC patients.

## Data Availability

Not applicable.

## Abbreviations

HCC: Hepatocellular carcinoma
CoA: Converts acetyl-CoA
HMGCR: HMG-CoA reductase
SQLE: Squalene epoxidase
LDLR: LDL receptor
SOAT: Sterol O-acyltransferase
NPC1L1: Niemann–Pick C1-like 1
SREBP: Sterol regulatory element binding protein
SCAP: SREBP cleavage activating protein
INSIG: Insulin-induced gene
ABCA1: ATP-binding cassette transporter A1
ABCG1: ATP-binding cassette transporter G1
HSP90: Heat shock protein 90
FoxM1: Forkhead Box M1
XBP1: X-box binding protein 1
NPC: Niemann–Pick type C
GRAMD1A: GRAM structural domain-containing protein 1A
LXRs: Liver X receptors
RXR: Retinoid X receptor
LXRE: LXR-responsive element
HBX: HBV X protein
SR-B1: Scavenger receptor B1
EMT: Epithelial–mesenchymal transition
